# Love and Infidelity: Causes and Consequences

**DOI:** 10.3390/ijerph20053904

**Published:** 2023-02-22

**Authors:** Ami Rokach, Sybil H. Chan

**Affiliations:** 1 Department of Psychology, York University, Toronto, ON M3J 1P3, Canada; 2 Faculty of Education, Western University, London, ON N6A 3K7, Canada

**Keywords:** love, infidelity, affairs, attachment injury, trauma, COVID, healing

## Abstract

This is a narrative review addressing the topic of romantic infidelity, its causes and its consequences. Love is commonly a source of much pleasure and fulfillment. However, as this review points out, it can also cause stress, heartache and may even be traumatic in some circumstances. Infidelity, which is relatively common in Western culture, can damage a loving, romantic relationship to the point of its demise. However, by highlighting this phenomenon, its causes and its consequences, we hope to provide useful insight for both researchers and clinicians who may be assisting couples facing these issues. We begin by defining infidelity and illustrating the various ways in which one may become unfaithful to their partner. We explore the personal and relational factors that enhance an individual’s tendency to betray their partner, the various reactions related to a discovered affair and the challenges related to the nosological categorization of infidelity-based trauma, and conclude by reviewing the effects of COVID-19 on unfaithful behavior, as well as clinical implications related to infidelity-based treatment. Ultimately, we hope to provide a road map, for academicians and clinicians alike, of what some couples may experience in their relationships and how can they be helped.

## 1. Love and Infidelity: Causes and Consequences

Grøntvedt et al., opined that “it is hard to imagine romantic and committed relationships devoid of transgressions of some kind. Despite the best intentions not to cause any harm or disappointment to one’s partner, breaking rules and promises are largely inevitable in long-term relationships” [1]. While some transgressions may be trivial and easily forgiven and forgotten, those involving betrayal may have significant effects on the relationship. It seems that any form of infidelity from either party may have potential to instigate a breakup [2]. In fact, research across 160 cultures revealed that spousal infidelity is the most common reason for a breakup [1]. Infidelity may not only have a destructive impact on the relationship, which may lead to separation or divorce, but may negatively affect the partners’ overall emotional wellbeing, leading to enhanced depressive symptoms andlowered self-esteem [3]. However, comprehensive reviews encompassing many aspects of infidelity (e.g., distinctions between emotional and sexual affairs, gender differences to extradyadic behaviors, the impacts of infidelity-based trauma) are sparse in the literature. As such, the following paper presents a “narrative review” of research related to the causes, consequences and reasonings of infidelity in adult romantic relationships.

## 2. Methods

We chose to include a mixture of qualitative and quantitative peer-reviewed research that directly addressed the subject of sexual and emotional infidelity, as well as infidelity-based trauma, as major variables of investigation for the purposes of this review. We focused on including work from a diverse collection of scholarly journals ranging from notable to lesser-known databases. We began our research by examining current issues from highly ranked journals in the fields of marriage and family therapy, sex therapy and couples counseling from the past 10 to 12 years. These journals included *Sexual and Relationship Therapy, Journal of Marital and Family Therapy, Journal of Family Psychology, Journal of Marriage and Family, Journal of Couple and Relationship Therapy, Journal of Sex and Marital Therapy* and the *Journal of Sex Research*. Following this, we reviewed several major social science databases, including ERIC, PsycINFO, PubMED and Google Scholar, using the following terms: “emotional infidelity”, “sexual infidelity”, “relationship trauma”, “extradyadic relationships”, “extradyadic behaviors”, “infidelity-based trauma”, “extramarital affairs” and “romantic betrayal”. We also examined the references of these articles and selected those that fit the criteria described above.

## 3. Defining Infidelity

While considerable research has been carried out on the topic of infidelity, little agreement exists regarding its definition. Drigotas opined that infidelity occurs when a person feels that their partner has violated the relationship norm by interacting with someone who is not a part of their relationship [4]. However, Blow and Hartnett defined infidelity as “…a sexual and/or emotional act engaged in by one person within a committed relationship, where such an act occurs outside of the primary relationship, and constitutes a breach of trust and/or violation of agreed upon norms (overt and covert) by one or both individuals in that relationship in relation to romantic, emotional or sexual exclusivity” [5]. Reviewing both definitions, a distinction needs to be made between sexual and emotional infidelity and its newer concepts related to inappropriate online and offline behavior, which we will address later [6].

Generally, infidelity is defined as any type of secret emotional, sexual or romantic behavior that violates the exclusivity that romantic relationships have by definition. However, there are varied definitions of infidelity, which can be divided into subtypes of sexual, emotional, combined (sexual and emotional) and internet infidelity [7]. Examples of the various (and sometimes contradictory) definitions, can be gleaned from Bernard who believed that partners who failed to love, honor and support their partners were engaging in infidelity, since they did not honor their vows to remain with their romantic partner [8]. In contrast, Pittman and Wagers held a different position and maintained that the hallmark of infidelity involves the secrecy and concealment of behaviors with an individual outside of the committed relationship [9]. Thompson had a more comprehensive view of infidelity, and postulated that infidelity occurs if: (a) the extradyadic behavior is not condoned by one’s romantic partner, (b) that behavior occurs outside of the primary relationship and (c) the behavior can be described, such as intercourse, flirting, etc. [10].

Sexual infidelity was defined by Leeker and Carlozzi as “the occurrence of sexual involvement with a third party that violates the ground rules established by the couple (e.g., kissing, fondling, oral sex, vaginal sex, anal sex)” [11].

Emotional infidelity was seen as “the occurrence of emotional involvement with a third party that violates the ground rules established by the couple (e.g., trusting another, sharing your deepest thoughts with another, falling in love in another, being vulnerable with another, being more committed to another, spending more money on another)” [11].

Research that explored which type of infidelity, sexual or emotional, would be more upsetting found that men were more distressed by sexual infidelity, while women were more upset by emotional infidelity [12,13] Research which addressed the reactions of lesbian and heterosexual women and gay and heterosexual men to infidelity found that for all four groups, emotional infidelity was more distressing than sexual infidelity [11]. Cramer et al., fund that women perceived emotional infidelity as more upsetting than men did, and the explanation provided by them was that women believe that men are not able to maintain sexual faithfulness in their relationships, but will still remain emotionally loyal to their spouses regardless [14].

Leeker and Carluzzi explored how sexual orientation, love and infidelity expectations might affect the reaction towards emotional and sexual infidelity [11]. Their study involved 296 individuals: 72 lesbians, 114 heterosexual women, 53 gay men and 57 heterosexual men, who were older than 18 years of age and who indicated that they were currently involved in a committed romantic relationship. They found that sex and sexual orientation were significant predictors of general distress, anger, anxiety, jealousy, humiliation, in response to both emotional and sexual infidelity. Commitment was predictive of distress and anger in response to emotional infidelity, while sexual infidelity aroused distress and anxiety.

Addressing the various types of infidelity, emotional infidelity includes the development of deep, intimate feelings for an extradyadic partner, while sexual infidelity refers to engaging in sexual behavior with that person. Those who engage in both emotional and sexual behavior are said to be involved with composite infidelity, while internet infidelity is carried out (at least initially) virtually/online [7]. Other researchers have employed even narrower definitions of infidelity by focusing on specific behaviors such as spending time with another individual and going on romantic dates, engaging in kissing, fondling, or even sexual intercourse, suggesting that they all constitute unfaithful behaviour [5,15].

Differences between the various types of infidelity were also observed in the work of Guitar et al., who reported that emotional infidelity is more complex than sexual infidelity [16]. Three hundred and seventy-nine undergraduate students provided their interpretations of emotional and sexual infidelity, which were later categorized into themes for content analysis. Participants’ responses indicated that emotional infidelity included themes such as love and betrayal along with sexual infidelity and/or intentions to have sexual relations with someone outside the pair bond. Particularly, women saw emotional infidelity as carrying the potential of later sexual betrayal in such partnerships. This suggests that the nuances involved with conceptualizing emotional infidelity may surpass the conditions needed to fulfill sexual infidelity, and that these differences may be most salient when observing differences across genders.

In fact, research has shown that men appear to hold more permissive attitudes towards extramarital sex than women do [17]. They also reported experiencing greater levels of stress related to the sexual infidelity of their partner, whereas women react more negatively to emotional infidelity than men [3]. However, women also seem to consider more behaviors as infidelity compared to men in both offline and online spaces [3].

Moreover, shared opinions regarding what specific behaviors are considered as unfaithful in nature have also been identified in the literature. For example, work by Bozoyan and Schmiedeberg found that extradyadic intercourse was regarded as infidelity [3]. Kissing someone who is not one’s partner was also reported as infidelity, especially if emotional involvement was part of it. The results of their research point to a perception of sexual infidelity as more distressing than emotional infidelity. However, women tended to judge behaviors as being unfaithful slightly more strictly than men, which is in line with other research in the existing literature [17]. Despite this, it appears that overall gender differences regarding the prevalence of infidelity have been shrinking over the past few decades [18].

### 3.1. Measuring Infidelity

Whitty and Quigley constructed a survey which aimed to explore what would upset participants from a list of several described situations [19]. Next, drawing from Harris and Christenfeld’s work, participants were then asked how they would feel if their partner was unfaithful and was in love with someone else [20]. Sabini and Green relied on Buss et al.’s much-utilized approach and described a situation where the partner of the participant was having deep emotional or sexual involvement with someone else [21,22]. Participants were asked to describe how they would feel in such a situation. They found that both, men and women, saw a partner’s emotional involvement as a more threatening sign of their partners’ leaving than when there was only sexual involvement.

In their study on infidelity, Leeker and Carlozzi utilized Cramer et al.’squestionnaire, in which participants rated the likelihood of their partner engaging in each item with a third party, on a seven-point Likert scale, aiming to identify their reactions to emotional vs. sexual intimacy [11,14]. Another measure which was utilized in Leeker and Carlozzi’s study was continuous emotion ratings [13]. These ratings served as the dependent variable in their study, assessing how angry, anxious, jealous and humiliated each participant felt in response to infidelity in their romantic relationship.

### 3.2. Perspectives on Infidelity

Symons and Buss were the first to view infidelity from an evolutionary perspective [23]. They opined that women are more likely to be affected by emotional infidelity rather than by sexual infidelity due to the fact that women carry the fetus and give birth. Thus, they are more threatened by the emotional betrayal of their romantic partner, for whom they count on to provide for the developing family unit. Men on the other hand are more threatened by sexual infidelity. Fisher et al., observed that for thousands of years, women depended on men to provide their food, shelter and safety, and this is the reason why they are more hurt by emotional infidelity which may threaten the partner’s commitment [24]. Men, however, are more threatened by sexual infidelity, as evolutionarily, they were not sure whether the child was theirs (versus the mother who carries her own child) and did not want to protect, feed and care for someone else’s offspring. This was referred to as jealousy as a specific innate module (JSIM). While the evolutionary perspective is the most accepted one, the social-cognitive perspective was proposed as an alternative to JSIM, and maintained that jealousy is not a simple module but includes several different feelings, each triggered by a different aspect of the jealousy-provoking situation. Anger was identified as a major component of the response to infidelity [25,26].

## 4. Distress Related to Emotional and Sexual Infidelity

### 4.1. The Role of Adult Attachment on Infidelity-Based Trauma

Colloquially dubbed as a “theory of trauma”, attachment theory was originally developed by John Bowlby to describe the different forms of emotional attachment (i.e., secure, anxious–ambivalent, avoidant, disorganized) and subsequent attachment behaviors that exist between a mother and infant [27]. As the child’s cognition begins to mature, they begin to develop internalized expectations, or internal working models, about how they should behave with their caregivers and how their caregivers should comfort them during times of distress or separation [27]. Overtime, the child learns how to perceive, process and resolve stressful events that involve their caregiver which ultimately inform their early understandings of attachment and later romantic relationships in adulthood [28].

However, parallels to this evolutionary behavioral system from infancy become prevalent as early styles and characteristics of attachment emerge in response to an unfaithful, romantic affair amongst committed partners [29]. Johnson et al., compares the harsh emotional pain experienced by victims of infidelity to the same attachment injuries as an infant separated from their mother [28]. Attachment injuries refer to traumatic interpersonal experiences which violate an individual’s internal representation of another as a trustworthy and reliable base for support [29]. The traumatic reactions caused by infidelity emulate behaviors and attitudes seen in a disorganized attachment style as immense emotional, psychological and cognitive dysregulation is evident amongst these afflicted romantic partners [5,30]. This includes reports of developing lower self-esteem, self-confidence, a lack of trust in others and a strong fear of abandonment in future romantic relationships [31]. Hazan and Shaver conclude that the subversive impact of infidelity harms the individual’s ability to be open to future romantic pursuits as the betrayal of a loving, secure partner is everlasting [29].

Reactions to infidelity vary significantly according to each person. Interpretations of infidelity are often based on an individual’s perspective of what they want to perceive from the event. The unfaithful affair may be interpreted as a threatening message or a conciliatory one; a process that is commonly referred to as causal attribution [32]. It would, consequently, be beneficial to enlighten couples prior to their long-term commitment—especially those whose attachment is not secure—that infidelity need not to “destroy” their trust in their partner, and that healing may occur, despite the traumatic event. Relationship satisfaction may also affect how people process and interpret these transgressions. Although, less satisfied partners may perceive infidelity as more threatening to the relationship, which may enhance the chances of relational dissolution. Others, on the other hand, may forgive the transgression and continue with their relationship [1].

### 4.2. Emotional Reactions to Infidelity

Infidelity can lead to emotional dysregulation for both victims and perpetrators of extradyadic behaviors. Specific emotional manifestations of infidelity-based trauma include feelings of extreme anger, betrayal, insecurity, rage, shame, guilt, jealousy and sadness [24,31,33,34,35,36,37,38,39,40].

Depressive symptoms following the disclosure of an affair are commonplace for victims of infidelity [30,33,38,41]. Women who had experienced threats of marital dissolution or of their husband’s infidelity were six times more likely to be diagnosed with a major depressive episode than those who had not experienced either of those events [42]. These women were also more likely to report heightened symptoms of nonspecific depression and anxiety [42]. Research by Lonergan et al., further supports these findings, as their participants demonstrated clinically significant scores of psychological distress which was associated with intrusive images, memories and rumination about their previous unfaithful relationship(s) [38].

Jealousy is the most frequently experienced emotion in response to discovering spousal infidelity. This mechanism was acquired by humans thousands of years ago and often occurs in combination with anger, insecurity, rejection, fear, betrayal, paranoia, depression, loneliness, confusion, envy and resentment, as well as PTSD [21,30]. Intense feelings, such as the ones mentioned, may trigger aggressive behavior, which may be expressed towards one’s spouse; this is the leading cause of homicide in the United States according to Leeker and Carlozzi [11]. It is evident that the impact of infidelity can have dangerous outcomes for those afflicted by this type of betrayal.

Future research may address the issue of intense emotions in light of romantic betrayal, aiming to find a method that the betrayed could employ to control those negative feelings. Thus, preventing that distress from overcoming their wishes regarding the union (assuming that they would want the union to continue).

### 4.3. Predictors of Emotional Reactions to Infidelity

Sex and sexual orientation were shown to be significant predictors of general distress, anger, anxiety, jealousy and humiliation in response to both emotional and sexual infidelity. Commitment was predictive of distress and anger in response to emotional infidelity, while sexual infidelity aroused distress and anxiety. Interestingly, Leeker and Carlozzi did not find an association between the influence of the three components of love on emotional responses [11]. Interestingly, neither passion or expectations about the likelihood of a partner committing sexual or emotional infidelity were able to predict emotional responses to either emotional or sexual infidelity. Generally, they concluded that women and heterosexuals are significantly more distressed by a current partner’s sexual or emotional infidelity in comparison with their male, lesbian and gay counterparts. As is intuitively apparent, those with greater commitment to their partners are more likely to be distressed and angered by a partner’s emotional infidelity, while those who feel that their union connection is less intimate will be more distressed and anxious by a partner’s sexual infidelity. Lastly, regardless of how passionate the relationship is, just imagining one’s partner being involved in infidelity evokes strong, negative emotions. Another interesting finding of the study was that gender was not predictive of jealousy in response to sexual infidelity.

Heterosexual men often report more distress in response to sexual infidelity than heterosexual women, although heterosexual women, lesbian women and gay men tend to report similarly high levels of distress to emotional infidelity. Apparently, it was found that sexual orientation was a significant predictor of emotional reactions to emotional and sexual infidelity. Commitment was positively correlated with distress and anger in the face of emotional infidelity, but not the sexual type. When there was less intimacy in the relationship, it was predictive of distress and anxiety in response to sexual infidelity, but not emotional infidelity. Leeker and Carlozzi opined that it is possible that having less of an emotional bond decreases the betrayed partner’s sense of safety and security when faced with a partner’s sexual infidelity, which may result in lowered distress and anxiety [11]. Notably, it is difficult to speculate why these emotions were felt in response to one infidelity type and not the other.

### 4.4. Effects of Sex and Sexual Orientation on Emotional Reactions to Infidelity

In Leeker and Carlozzi’s study, gender and sexual orientation did not significantly interact to elicit emotional responses to sexual and emotional infidelity. Women, regardless of their sexual orientation, reacted more strongly to both types of infidelity than men. Women’s reactions to emotional infidelity were similar to those of men, while they were angrier than men in the face of sexual infidelity. When faced with sexual infidelity, women were almost as humiliated as they were anxious and jealous, whereas men were much less concerned with humiliation. Both women and men were more distressed by sexual infidelity than emotional infidelity overall [11,43,44].

In the Leeker and Carlozzi study, women and men agreed that sexual infidelity mostly elicited anger, followed by anxiety and jealousy [11]. Additionally, these researchers found that heterosexuals’ scores were also significantly higher than lesbian and gay individuals’ scores, but no significant sexual orientation differences were found between emotional and sexual infidelity. Evolutionary theory would explain this result by suggesting that lesbian and gay people should not be as affected by infidelity compared to heterosexuals, since infidelity by same-sex partners does not pose the evolutionary threats of raising another man’s child or losing a male partner’s resources to another woman [45].

### 4.5. Effects of Infidelity Type on Emotional Reactions

Among all participants of the Leeker and Carlozzi study, sexual infidelity elicited significantly more intense emotional reactions than emotional infidelity, with significant differences in distress, anger and humiliation [11]. Sexual infidelity elicited significantly more anger than the other emotions. The researchers also found that emotional infidelity elicited significantly more anxiety and jealousy than anger and humiliation. Sexual infidelity, on the other hand, elicited significantly more anger than all other emotions and may, thus, reflect the common viewpoint that sexual infidelity is preventable and intolerable, whereas emotional infidelity is perceived as less controllable [46].

Seminal research by Buss et al., asked college students to imagine their romantic partner being engaged in a deep emotional attachment with another person or imagine their partner enjoying passionate sexual intercourse with that person [21]. Participants were then asked which upset scenario them more. Results found that 60% of men believed sexual infidelity to be more stressful, whereas only 17% of women felt that way. This is in line with evolutionary theory, which state that men and women react differently to the two different transgressions as a result of sexually dimorphic selection pressures [47].

### 4.6. Understanding Infidelity Victimization in the Context of Trauma and Stressor-Related Disorders

The diagnostic category of “trauma and stressor-related disorders” is a new addition to the fifth edition of the *Diagnostic and Statistical Manual for Mental Disorders* (DSM-5) which lists external environmental stressors as an etiological factor for various mental illnesses [48]. For PTSD, the stressor must involve exposure to or experience involving actual or threatened death, serious injury or sexual violence (e.g., Criterion A) [48]. In contrast, the stressors needed to diagnose adjustment disorder [AD] can include those that fall into “everyday” normal life such as job loss, the death of a loved one or divorce [38,48]. According to Maercker and Lorenz, the similarities of both conditions can be traced back to cognitive distortions surrounding safety and trust which are developed from the maladaptive memories of a traumatic event [49]. They suggest that these memories are often combined with negative appraisals about the traumatic incident to radically impact an individual’s opinion of the world and themselves as dangerous, damaging and destructive.

Several authors have cited the emotional, cognitive and behavioral reactions to infidelity as evidence to support its status as a traumatic experience that is comparable to PTSD, despite AD being a better diagnostic fit [38,50,51]. Both disorders share similar psychophysiological symptoms that are typically demonstrated in cases of infidelity, including feelings of elevated anxiety, hyperarousal, rumination, intrusive flashbacks, emotional dissociation and depression [50,52]. However, an important distinction that should be stressed is of the diagnostic criterions needed to diagnose PTSD in the DSM-5. For example, research by Steffens and Rennie and Laaser et al., found that infidelity victims met all the criteria for PTSD apart from Criterion A [41,53]. Similar results were shown in work by Roos et al. and Gordon et al., which found that victims of romantic betrayal experienced clinical levels of PTSD symptomology that included high levels of depressive symptoms and stress [51,54]. These findings suggest that victims of romantic betrayal do experience significant psychological and emotional distress, but not due to the trauma of direct or threatened exposure to deadly circumstances, as needed to fulfill Criterion A for PTSD [48]. Therefore, these symptoms may be better understood within the diagnostic context of AD, rather than in PTSD.

It is therefore suggested that attachment-based trauma, as seen in extradyadic affairs, should be regarded as a valid traumatic experience, but should also be nuanced and critically distinct to that of PTSD trauma [30,35,55]. Some clinicians argue that framing the experience of infidelity as a form of trauma may facilitate greater emotional recovery for its victims, thus, demonstrating the benefits of validating such an emotionally distressing event [56,57]. Therefore, conceptualizing infidelity as its own unique traumatic experience within the lens of AD may help to accurately capture the impact of these situations while shedding light on the potential overdiagnosis and reliance of the PTSD label [49,58,59].

### 4.7. Physical Health Consequences of Infidelity-Based Trauma

Few studies have examined the relationship between infidelity-based trauma and its subsequent physical health consequences, although immediate physical reactions shortly following the discovery of these affairs have been cited by some researchers. For example, work by Lonergan et al., found that infidelity victims reported persistent somatic symptoms such as insomnia, weight loss, difficulty with concentration and a lack of appetite and libido immediately after experiencing romantic betrayal [38]. Another study conducted by Roos et al., found that undergraduate students experiencing infidelity as victims of romantic betrayal reported having difficulty breathing, bodily trembles, extreme nervousness and a racing heart when recalling their previous relationship [51]. This is further supported by findings from Shackelford et al., which found that female participants reported greater symptoms of nausea and physical illness when asked to imagine their partner as unfaithful under experimental settings [40]. Ultimately, it is suggested that further work should be performed to examine the lasting physical effects of infidelity-based trauma to promote preventative care for those involved in these relationships.

### 4.8. Suicidality and Infidelity

Infidelity may feel like an unstoppable problem which may invoke thoughts of suicidal ideation and suicidality among vulnerable individuals [60,61]. An article by Snyder et al., described how these issues may impact both perpetrators and injured partners of an affair [62]. For the injured party, fluctuating feelings of rage, powerlessness, abandonment and victimization may leave them shaken and unable to support themselves after learning about their partner’s betrayal. This may lead to suicidal ideation. However, perpetrators of infidelity may experience similar emotional reactions after the discovery of their affair, such as depression, acute anxiety and suicidality. This is believed to occur persistently following threats of divorce or marital separation following disclosure [62].

To date, only one study by Stephens has closely examined the relationship between suicidal behavior and relationship-related distress such as infidelity [63]. A community sample of 50 women with histories of previous suicide attempts were asked about their intimate relationships with men in relation to their suicidal behaviors. It was found that partner infidelity, along with battering, “smothering love” and denial of affection, were the most prominent themes that lead to suicidality and suicidal ideation. Interestingly, Stephens also asserted that age may be a confounding variable for this topic as well [63]. Younger participants reacted to specific negative events in their romantic relationships by attempting suicide, while older participants would do so in response to long-term conflicts with their partners. This may suggest that the threshold for suicidality in those afflicted by infidelity is different for individuals depending on their age and range of experiences related to love.

Similarly, research by Martin et al., further supports the relationship between infidelity and suicidal behavior in their work examining the role of marital status, life stressors and communication regarding suicidality in U.S. Air Force personnel [64]. Researchers examined 100 decedents who died by suicide and examined their social supports (e.g., communication with friends, family and coworkers), medical and fiscal records (e.g., personnel files, finances, mental health details), toxicology and autopsy reports and evidence from the death scene (e.g., suicide notes) to gather a comprehensive understanding of the conflicts that contribute to suicide. Among this sample, 9% of suicide completers were found to have experienced the infidelity of a spouse within 24 h prior to their passing. Martin et al., also observed that 5% of decedents had committed infidelity within this time frame as well [64]. Findings from this study highlight the rapid and deadly risks of infidelity disclosure in precipitating suicidal behavior for both victims and perpetrators of romantic betrayal.

These limited studies show that further research is necessary to examine specific factors that drive suicidality in certain victims of infidelity. The importance of this topic warrants greater investigation into how potential influences such as age, personality and relationship duration impact infidelity-based suicidality.

### 4.9. Why Do People Think They Get into Affairs?

Selterman et al., wanted to understand how those who were involved in infidelity feel, think and behave, and suggested that all of these factors are affected by their motivation to have extramarital affairs [65]. Their findings suggest that there may be meaningfully different infidelity typologies characterized by both different underlying motivations, different relational processes and different behavioral outcomes. Thompson’s deficit model of infidelity suggested that relationships which are not optimal and are characterized by low satisfaction, high conflict and a lack of good communication play a significant role in the causal factors leading to infidelity [10]. Like other models and theories, this model sees infidelity as a symptom of deeper underlying relational difficulties that the couple is struggling with. In their study, Selterman et al., explored the infidelity of 495 participants, including 259 women and 213 men who had significant variability in relationship length, ranging from 1 month to 28 years [65]. Results indicated that while almost all participants engaged physically with their affair partners, only 53% had intercourse with them. Men were more likely to report engaging in these sexual behaviors. Those motivated by sexual desire, and seeking love and variety, reported greater sexual satisfaction with their affairs. On the other hand, those motivated by situational factors were less sexually satisfied with the affair, which was also short lived, in contrast with those in long-term committed relationships [66,67].

Selterman et al., found eight different variables which related to infidelity motivation. These included things such as feeling angry at a partner’s behavior; wanting more sex than is available in the primary relationship; wanting more intimacy and love than is available to them; having low commitment to the relationship; wanting greater autonomy; clouded judgement due to situational factors, such as stress; feeling mistreated or neglected; and wanting a greater number of sexual partners [65]. Consistent with the deficit model of relationship infidelity, they found that motivations related to a lack of love and neglect predicted participants’ reported intimacy with affair partners, such as expressing their love verbally in “I love you” statements, public displays of affection and engaging in longer affairs, while situational motivation was inversely associated with these experiences. The authors opined that when people feel emotional shortfalls in their primary relationships, they may search for a deeper quality of romantic connection which includes more intimacy in their affairs to compensate for the insufficient intimacy experienced with primary partners.

Furthermore, emotional closeness to their primary partners was negatively associated with the emotional satisfaction which people involved in affairs experienced [65]. In some instances, people become involved in affairs to hurt their partner. They are usually angry, score lower on commitment and experience a lack of love in their relationship. In light of the devastating effects of affairs, it is possible that while some participants wanted their primary partners to suffer, others had no intention to hurt their partner or terminate the relationship [68]. Commitment affected the post-affair contact that people maintained with their affair partners; those who had a higher level of commitment, versus those who did not, did not maintain contact with their affair partners. Focusing on one’s partner and the relationship may enhance personal and relational growth following an affair, while if that is missing, the relationship may not survive an affair. People who lacked love, appreciation and sexual desire in their primary relationship are more liable to leave it and establish a primary relationship with their affair partner [65].

## 5. Infidelity in Marital Relationships

The scientific literature points to the occurrence of what is variously labeled infidelity, extradyadic involvement, unfaithfulness, affairs, stepping out, cheating or some other synonym indicative of secret romantic activity with a secondary partner while in an exclusive romantic relationship. This secretive activity can range from emotional involvement all the way to penetrative sex. Estimates suggest that infidelity occurs in about a quarter of all marriages, and at the beginning of the 21st century, a dramatic increase in infidelity of the oldest cohort of men (ages 65–90) was noted [18].

Infidelity causes grief and relational problems to the individual, the couple and even their offspring. It was found to be associated with depression, anxiety and even PTSD, leading to divorce [42,69,70]. Additionally, infidelity was linked to domestic violence and increased exposure to sexually transmitted diseases [21,71].

### 5.1. Factors That Increase Infidelity

#### 5.1.1. Demographics

While early research suggested that men are more likely to commit infidelity than women recent work has suggested that the gender gap is narrowing [56,72]. A study observing the relationship between religion and infidelity found that non-religious people report more cases of infidelity than religious ones [73]. Education has also been shown to be positively associated with infidelity, in that those with higher education are more likely to engage in infidelity than the less educated, often depending on other factors in their lives. Individuals with higher incomes are also more prone to engage in infidelity, although, this may simply be because their professional and personal lives include more opportunities to engage in extradyadic relations. About half of all those who engaged in infidelity met their extradyadic partner at work [74].

#### 5.1.2. The Individual

Personal characteristics such as neuroticism, prior history of infidelity, number of sex partners before marriage, psychological distress and an insecure attachment orientation, as well as permissive attitudes toward sex, have been positively associated with infidelity [75,76,77]. Coming from a family where infidelity was present also increases the risk of one being involved in infidelity [7].

When not caused by marital conflict or low marital satisfaction, infidelity may be associated with opportunity and permissive values. For instance, Treas and Giesen found an increased likelihood of sexual infidelity among men and women with stronger sexual interest levels [78]. Some research used the Big Five personality traits and found extraversion, high neuroticism, low conscientiousness and high psychoticism to be positively correlated with engaging in infidelity [76,79]. The dual control model of sexual response suggests that one’s sexual behavior depends on the balance of sexual desire and inhibition; inhibition may be related to fear of performance failure or of possible consequences related to extradyadic sexual relations [80]. A number of studies have shown that the propensity for sexual excitation is related to sexual responsiveness, sexual desire levels, sexual compulsivity and a lifetime number of casual sexual partners. Sexual inhibition may be adaptive, but high levels of it may lead to sexual dysfunctions, while low levels may result in increased risky sexual behavior [81].

In their 2011 study, Mark et al., found that up to 22% of people engaged in extradyadic relationships [82]. They found that perceived sexual compatibility and happiness in a relationship were significant predictors of infidelity in women, while age, marital status and the importance of religion did not significantly affect one’s proclivity for affairs. They also found that a stronger tendency to lose one’s sexual arousal when facing possible risks serves as a protective effect for engaging in infidelity. Interestingly, they found that experiencing sexual problems in their extradyadic relationship was less threatening for individuals with arousal difficulties. The authors propose that perhaps these individuals are less concerned with their sexual performance with a partner to whom they are not emotionally committed to or one they have been with for a long time. As can be expected, they found that higher levels of sexual excitation were associated with increased sexual risk-taking behaviors, particularly in men. Women were found to be more likely to engage in infidelity when they were dissatisfied in their relationship or felt that they were sexually incompatible with their partner, which may point to the interconnection of sexual and relationship factors in increasing the possibility of infidelity. In other words, if a woman is unsatisfied with her current relationship, she may seek intimacy and closeness somewhere else. An interesting finding of Mark et al.’s study was that sexual excitation did not predict involvement in infidelity for women [82]. That may support the notion that women’s sexual infidelity is less motivated by sexual needs, arousability or desire, while in men, this is often not the case.

#### 5.1.3. Relationships

Decreased satisfaction in a present relationship is closely related to infidelity amongst married people [83]. When commitment is not central to the relationship, that too contributes to infidelity [4]. Interestingly, cohabitation before marriage was found to be positively associated with infidelity [84].

#### 5.1.4. Context

The gender gap in infidelity of married couples is ascribed to women’s increased presence in the working world. There, the woman spends many daily hours working closely with the opposite sex and interacting about issues and topics that they both seem to value. Moreover, when one spouse works out of the home and the other stays at home, the chances of infidelity increase [85]. In the last twenty years, the internet has provided increased opportunity for infidelity. Up to 30% of internet users go online for sexual purposes, and up to two-thirds of them engage in offline sexual intercourse with their online partner [86].

#### 5.1.5. Marital Deception

Dew et al., explored two kinds of marital deceptions: financial marital deception (FMD) and extramarital infidelity (EMI) [87]. EMI was well researched, while MFD was much less so. Interestingly, one may bring about the other. Social exchange theory (SET), which originated in social psychology’s interpersonal relationship area, and was pioneered by Thibault and Kelley, asserted the rewards, costs and expectations that partners have of their relationship, which may entice them to remain with their partner, modify the relationship or leave all together [88]. Nye observed that each spouse evaluates the “outcomes”, meaning the costs and benefits entailed in their marriage [89]. Then, the spouses compare the outcomes to those to which they expect to receive in that relationship, which are termed in SET, the comparison level, or CL; this will determine whether the spouse will remain in the marriage. If a spouse finds that his or her marital outcomes exceed the CL, they will be satisfied with the relationship and remain in it. However, if a spouse’s outcomes fall beneath their CL, they will become dissatisfied with the relationship and may seek to change or terminate it. This may lead to relationships out of the marital union, remaining in the relationship despite a lowered satisfaction level or leaving the relationship. Dissatisfied spouses will engage in what was termed “comparison level of the alternative” (CLalt), which may lead them to leave the relationship.

#### 5.1.6. Moral Commitment

To remain in the marriage was found to be negatively associated with EMI and possibly MFD. Most people, at least in Western countries, want marital fidelity and plan to avoid EMI. They want to behave in a way that upholds marital norms and/or their wedding vows to their spouse, and to remain loyal to them [90]. Personal dedication to one’s marriage, and the desire to make it succeed, is a type of commitment to the marriage, which is separate from moral commitment, since dedication is focused particularly on increasing the rewards and happiness of the couple [91]. Personal dedication, then, may make it less likely for marital dissatisfaction to occur when its outcomes fall below the CL. This may lead to a situation in which a spouse may be dissatisfied with the marriage, but viewing it as a long-term commitment will motivate them to invest in it and be less interested in alternative relationships, despite their present unhappiness [92]. Recent research about MFD and EMI found that personal dedication commitment (in the form of marital stability and trust of one’s partner) is negatively associated with MFD [87]. Additionally, personal dedication commitment is associated with a lower level of sexually unfaithful behaviors [93]. Religiosity was also found to be associated with a better marital relationship, since most religions hold marriage to be sacred and special. This may stem from religious peoples’ hesitation to violate something that they believe is sacred, particularly when they are part of a religious community which does not condone infidelity and unfaithfulness [94]. Dew et al., also found that those who engaged in minor EMI, such as flirting, had an increased likelihood of engaging in EMI with the person with whom they flirted, in addition to increased chances of engaging in MFD, since marriages that are growing in a positive direction provide less motivation to engage in EMI [93,95].

## 6. The Effect of Infidelity in Cyberspace

Social media sites are platforms where users generate and post their own content to create and maintain virtual relationships [96]. These platforms are very popular, as indicated by, for example, Facebook’s 2.45 billion active monthly users [97]. These popular platforms contribute to increased opportunities for infidelity [98]. In her research, Adam found that flirting or sexual behavior conducted via social media is indeed perceived similarly not only to cyber-sexual behaviors but also to physical sexual infidelity, which is similarly hurtful to romantic relationships [98].

### 6.1. Infidelity and COVID-19

When discussing infidelity, we would be remiss if we did not review research that assessed infidelity during the pandemic that swept the world just a short time ago. Gordon and Mitchell asserted that COVID increased the chance that people would be involved in infidelity, particularly in light of the stress that was brought about by the pandemic [99]. These challenges may potentially have resulted in lower relational and sexual satisfaction, which may justify—in the eyes of a partner—becoming involved in an affair [100]. While social distancing was practiced during the pandemic, and has consequently decreased the opportunities for physical contact with affair partners, the use of virtual apps to stay connected (e.g., Face Time, Zoom, and Skype) drastically increased during this time and may be more likely to be used to contact affair partners than prior to the pandemic [101]. Dating sites have flourished, and they have also been utilized as an opportunity to get involved in affairs [102]. Infidelity may have devastating consequences for the couple, and those discovered during the pandemic may have a greater possibility to cause negative consequences [99]. Anxiety and depression, which are known to follow the discovery of an affair, may be exacerbated due to the pandemic, which by itself is liable to cause such a reaction [103]. Significant financial loss, which occurred frequently due to closures of work sites and limited operations or unemployment, may also precipitate infidelity [103].

### 6.2. How Does COVID-19 Impact Affair Recovery?

The pandemic may have made recovering from infidelity more complicated. During that time, the couple’s access to healthcare resources and social support, such as their friends and confidants, was more restricted; thus, addressing the emotional injury that an affair caused was much more difficult [104]. Additionally, couples who focused on decreasing their anxiety and stress caused by the pandemic, dealing with financial concerns and spending their mental and emotional energies on struggling to survive during such a difficult time may have been less able to cope with difficulties caused by an affair [105]. Heightened emotional arousal resulting from both the pandemic and the affair may make it more difficult for couples to effectively regulate their emotions during this time, which may slow or even inhibit the healing process, as the couple may be more irritable and liable to strike out at the slightest provocation [106]. Additionally, while it is common for couples to take a break from each other (either by spending more time apart, or even moving to a different house for a period) after the discovery of an affair, the pandemic made such a break impossible due to strict rules related to social distancing. This may have seriously disturbed the healing time that such a separation provided [104].

Gordon and Mitchell observed that while communication is an important factor in healing, constantly discussing the affair and the details of the extradyadic relationship may be more harmful than helpful to healing, as partners may not yet be emotionally ready to discuss them [99]. Infidelity is a much-stigmatized phenomenon and responses such as shame, shock, anger, hurt and despair may result in constant friction at home. These intense emotions need space and time to be expressed and processed; being together 24/7 disallows this. Additionally, couples try to hide the affair from their children; this was especially true during the pandemic. Partners constantly being at home while dealing with infidelity and its aftermath means that these conflicts may have easily been overheard by the children, increasing their anxiety and familial stress. 

An important component of the recovery process is rebuilding the trust that was lost as a result of the affair. This is, usually, a slow process which requires a concerted effort on the part of both partners, which is often not linear [104]. One of the first steps in rebuilding trust is for the “offending” partner to stop seeing the affair partner, which may require changing where one goes to the gym, shopping, etc. The pandemic has changed all of this. On the one hand, the strict social distancing rules may have decreased the chance that affair partners would continue to meet, but on the other hand, various forms of virtual contact may have continued [106].

Gordon and Mitchell concluded their study by observing that “infidelity is a wrenching and devastating event that is difficult for couples to navigate even under the best of circumstances [99]. Experiencing this relational trauma during a global pandemic that is also traumatic with far-reaching social and economic consequences is even more overwhelming. This context can intensify and exacerbate normal emotional reactions to affairs and complicates efforts toward recovery. Couples will need to dig deep and intentionally build emotional resources to meet these challenges. However, all is not lost in this context and there is hope for couples’ recovery during this time. After many years of working with couples to deal with the discovery of an affair, we have found that couples can be astonishingly resilient…Thus, COVID-19’s vast and life-changing impacts can create added challenges and barriers in couples emotional and social lives, but as the cliche suggests, it also can create opportunities for immense growth for these couples and the clinicians who are trying to help them” [99].

### 6.3. Therapists Addressing Infidelity: Challenges and Attitudes

As infidelity remains one of the major causes of divorce, it is essential that therapists are trained to help couples deal with what can be a devastating personal and relational experience [107]. Irvine and Peluso explored therapists’ subjective experiences with treating affairs [108]. Professional guidelines, such as those of the American Association of Marriage and Family Therapy or the American Psychological Association, state that therapists are expected to practice competently when treating individuals or couples [109,110]. Given the complex and morally laden nature of affairs, therapists may confront challenges that can significantly impact treatment outcomes. Among those challenges, the therapist may experience countertransference and then over-identify with one partner, which will hamper their neutral position as a counselor [111]. Garza conducted a study that revealed that therapists’ attitudes toward infidelity can clearly influence their treatment decisions [112]. Specifically, therapists with more negative views towards infidelity guided the couple in reducing environmental risk factors (e.g., limiting Internet access) related to the affair, rather than addressing larger processes that impacted the couple’s presenting issue. Other struggles that therapists face when dealing with clients facing infidelity may involve their need to strike a balance between addressing the needs of both partners while exploring the underlying causes of the affair. Additional challenges that emerge from this process include having to establish trust and forgiveness between the partners healing from the emotional injury, which could resemble symptoms of post-traumatic stress disorder such as hypervigilance and increased distress [113].

Irvine and Peluso were interested in exploring a myriad of therapist-related factors that impact counseling for couples faced with infidelity [108]. These included influences such as the personal and professional experiences and histories of therapists who treated infidelity, and the challenges they faced when treating couples in these circumstances. They found that the specific experiences of the counselor directly impact the competency of treatment for those recovering from a romantic affair. Therapists who had attended infidelity training, held their license for more than 16 years, held a doctoral degree and were licensed to practice marriage and family therapy showed the highest levels of comfort, preparedness, effectiveness and confidence in treating infidelity [113]. In turn, four factors in couples were identified by Irvine and Peluso that negatively affect recovery from romantic affairs [108]. This included things such as the betrayal continuing while the couple was in therapy, an unwillingness to commit to therapy, continual blame and resentment towards each other without forgiveness.

Ultimately, further training related to the issue of infidelity is suggested for clinicians working with couples [108]. Research has shown that the vast majority of therapists have never received any courses on infidelity, and that this had hampered their perceived competence when treating such individuals [114] Several factors which may impede effective treatment delivery have been identified by Irvine and Peluso [108]. These factors included learning how to manage one’s countertransference reactions, knowing how to address trauma and manage emotional reactivity, possessing clinical experience and balancing needs that arise in the process of therapy.

## 7. Conclusions

Clearly, the consequential effects of infidelity vary widely according to the type of extradyadic behavior performed, in combination with the demographic and interpersonal factors of the people in question. Men and women react to emotional and sexual infidelity differently, as research suggests that women tend to judge more behaviors as unfaithful, while men hold more permissive attitudes towards extramarital sex [17]. This may be explained by evolutionary psychology as the genders’ attempt to protect their union and offspring. Additionally, the literature on infidelity has also shown that younger people express greater negative attitudes toward infidelity and more often perceive sexual behaviors as infidelity than older people [115]. This is further supported by the work of Varga et al., who observed that age may have a moderating effect on the gender differences concerning sexual versus emotional jealousy [116]. Researchers further suggest that individuals who are most likely to commit infidelity are more educated, wealthier and less tied to a religious faith [74].

Infidelity may not only have a destructive impact on the relationship leading to separation or divorce; it can also negatively affect one’s emotional wellbeing by enhancing depressive symptoms, highlighting low self-esteem and promoting remorse in the unfaithful party [3]. This type of attachment injury could impose psychological and emotional dysregulation for those facing these circumstances, which may emulate symptoms of conditions such as depression, anxiety and AD [28,30,38,41,51,53]. The impact of this life-altering event challenges the person’s sense of self, safety and trust in another who is supposed to be their “secure base” for love and adoration [29,31]. Thus, infidelity leaves some at risk of turning towards unhealthy coping mechanisms such as excessive drinking, drug use, unprotected sex, and suicidal behavior in response to their emotional pain [63,64,70,117].

Overall, it is clear that the implications of a romantic affair have a substantial impact on one’s life beyond their intimate relationships. Clinicians are encouraged to seek professional training when treating couples afflicted by infidelity and to be conscious about how their own moral and personal views related to the matter could impact their clients’ recovery from these circumstances [108]. Scuka suggests clinicians normalize the experience of infidelity for their clients, as this can serve as the first step in identifying realistic expectations for the healing process [118]. However, communication regarding the details of an extradyadic affair should be guided between partners, as Gordon and Mitchell stress the importance of being emotionally ready for those conversations [99]. Therefore, it is recommended that therapists should be mindful of integrating high levels of sensitivity, care and honesty in these sessions to facilitate appropriate closure for those impacted by infidelity.
